# Therapeutic Strategies and Clinical Outcomes of Infections Following Anterior Cruciate Ligament Reconstruction: A Narrative Review

**DOI:** 10.1111/os.70298

**Published:** 2026-03-18

**Authors:** Shaoli Zhang, Jue Gong

**Affiliations:** ^1^ Department of Sports Medicine Xinhua Hospital Affiliated to Dalian University Dalian China

**Keywords:** ACLR, postoperative infection, risk factors, treatment strategies

## Abstract

Anterior cruciate ligament reconstruction (ACLR) is a widely performed orthopedic procedure, yet postoperative infection, although rare, poses a significant threat to graft integrity and long‐term joint function. This review specifically focuses on therapeutic strategies for ACLR‐associated infections. Management strategies constitute the core of this review, centering on early surgical debridement, targeted antimicrobial therapy, and, in selected cases, graft retention or removal. Preventive and rehabilitative measures such as graft presoaking with vancomycin, strict intraoperative asepsis, and structured postoperative rehabilitation are also discussed. Long‐term functional outcomes are often suboptimal, emphasizing the importance of timely rehabilitation and individualized care. Rather than providing an exhaustive diagnostic review, we highlight therapeutic decision‐making and evidence‐based treatment pathways, supplemented by stratified comparisons of prospective and retrospective clinical studies. Ongoing research into biofilm‐targeting therapies is essential to optimize treatment protocols and minimize infection‐related complications.

## Graphical abstract

ACL reconstruction‐associated infections threaten graft integrity and joint function. This review summarizes patient‐related risk factors, approaches, and treatment strategies, with emphasis on early recognition and preventive measures such as vancomycin presoaking.

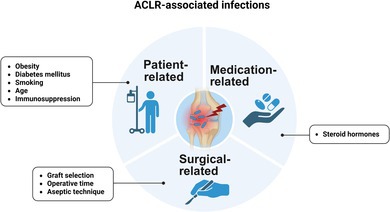

## 1 Introduction

Anterior cruciate ligament (ACL) rupture is one of the most common knee injuries encountered in young and physically active populations, particularly athletes [1, 2, 3]. ACL reconstruction (ACLR) has become the standard of care for restoring joint stability and enabling return to pre‐injury activity levels, with an annual incidence estimated at 74.6 per 100,000 individuals in commercially insured populations and ~40 per 100,000 in national registries such as Sweden's [4, 5]. While generally considered a safe and effective procedure, postoperative infection, though infrequent—reported between 0.1% and 2.44%—remains one of the most devastating complications, often leading to prolonged recovery, graft failure, cartilage damage, or permanent disability [6, 7, 8, 9]. Recent literature highlights increasing attention to ACLR‐related infections, which not only lead to poor functional outcomes and psychological distress—particularly in professional athletes—but also impose substantial economic burdens due to prolonged hospitalization, revision surgeries, and delayed return to sport [8, 10].

**FIGURE 1:**
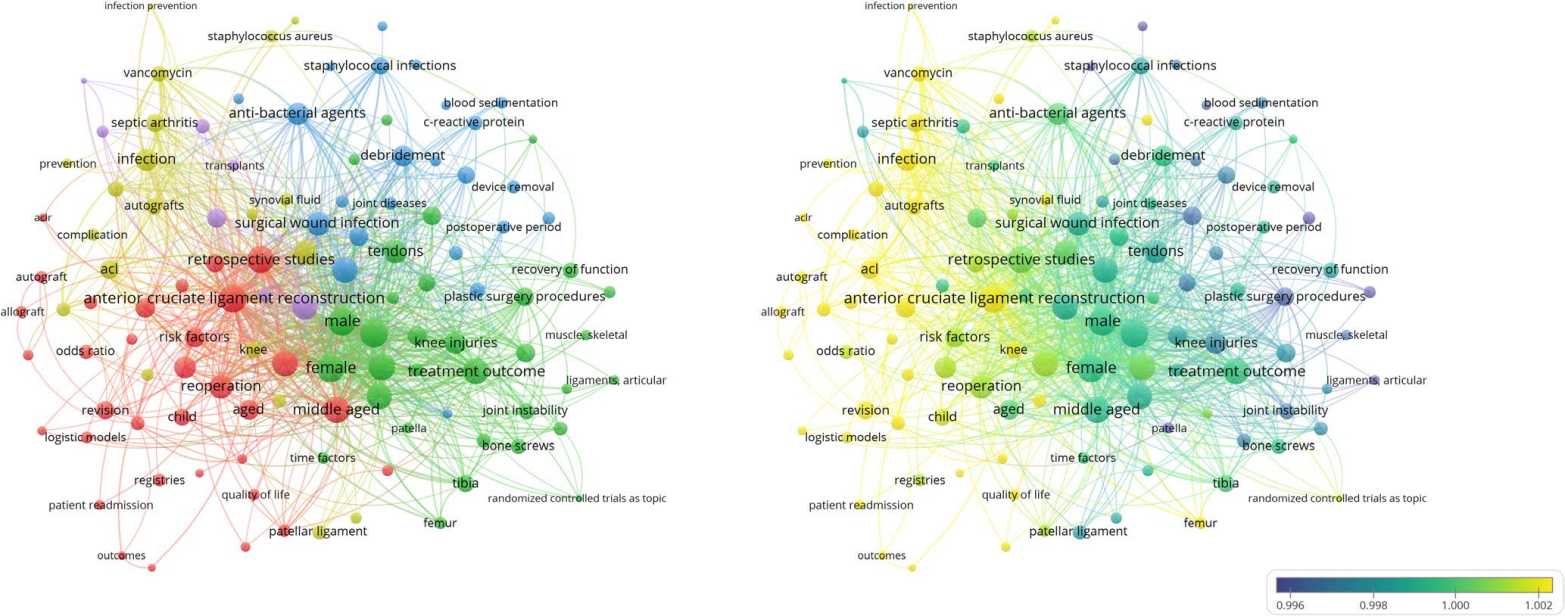
Keyword co‐occurrence map of PubMed publications related to anterior cruciate ligament reconstruction (ACLR) and infection.

Despite growing recognition of its clinical significance, existing studies remain fragmented across diverse aspects—such as risk factors, treatment approaches, and prevention strategies—without an integrated framework to guide comprehensive management. Among the emerging hot topics in this field, recent keyword co‐occurrence analysis of PubMed publications on ACLR and infection reveals concentrated interest in “Staphylococcus,” “Risk factors,” and “C‐reactive protein” (Figure 1), reflecting a shift toward exploring both microbiological and host‐related determinants of infection. Systematic reviews and meta‐analyses have begun to consolidate data from large cohorts, identifying high‐risk groups (e.g., males, obese patients, diabetics, those with prior corticosteroid use or previous surgeries), while underscoring modifiable surgical factors such as graft type, procedure duration, and vancomycin presoaking practices [11].

In light of these developments, the present review narrows its scope to therapeutic strategies for ACLR‐associated infections. By critically examining available clinical studies and stratifying evidence from prospective and retrospective research, this paper aims to provide an in‐depth, treatment‐focused reference to guide individualized decision‐making and optimize outcomes in patients with infection after ACL reconstruction.

## 2 Search Strategy and Selection Criteria

To ensure a rigorous synthesis of evidence, a systematic search was performed in the PubMed database up to February 2026. The search strategy employed keywords including ACLR, postoperative infection, septic arthritis, risk factors, postoperative complications, and treatment strategies. Inclusion criteria comprised peer‐reviewed clinical studies (RCTs, cohort, and case–control studies) and evidence‐based syntheses (systematic reviews and meta‐analyses) related to joint infection post‐ACLR, specifically focusing on therapeutic interventions and independent risk factors. Exclusion criteria were: [1] duplicate publications or studies without full‐text access; [2] conference abstracts or editorials; and [3] non‐human studies. Independent literature screening and data extraction were conducted to synthesize the current landscape of post‐ACLR infection management.

Through the systematic search, we identified and synthesized evidence focusing on the clinical management and determinants of infection after ACLR. The following sections detail the risk factors and therapeutic strategies identified in the current literature.

## 3 Risk Factors

### 3.1 Patient‐Related Factors

**FIGURE 2:**
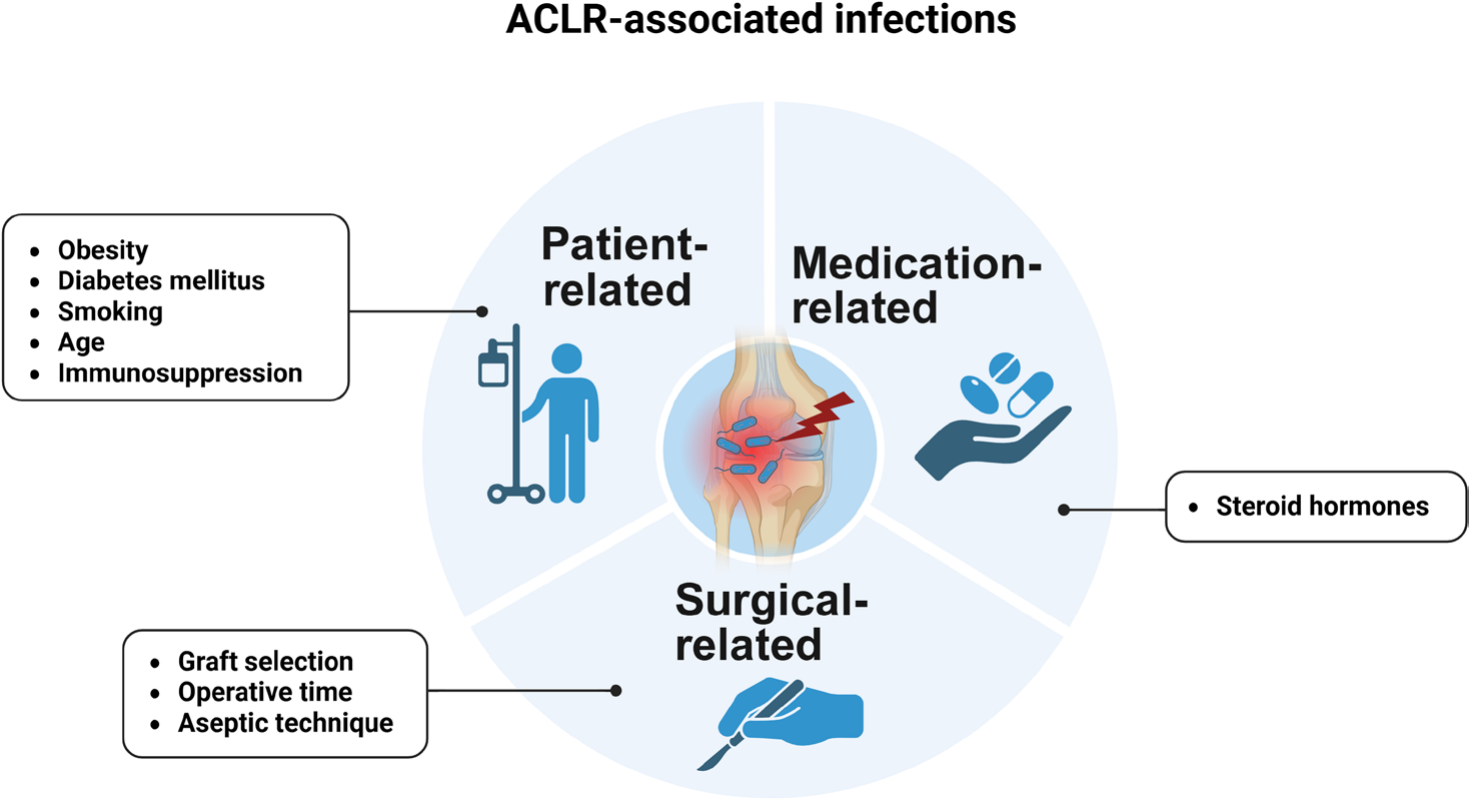
Patient‐related risk factors contributing to infection after anterior cruciate ligament reconstruction (ACLR).

Patient characteristics such as age, comorbidities, and lifestyle habits play a crucial role in postoperative infection risk after ACLR [12, 13] (Figure 2). Obesity triggers a pro‐inflammatory state via NLRP3 inflammasome activation and IL‐1β secretion, which impairs wound healing and compromises adaptive immunity through thymic involution [14]. In addition, diabetes mellitus induces immune dysfunction and delayed leukocyte recruitment via microvascular damage and hyperglycemia. In smokers, reduced vascular perfusion and tissue oxygenation hinder collagen synthesis [6, 15]. notably, smokers exhibit a significantly higher infection rate (2.0%) compared with non‐smokers [16]. Accordingly, comprehensive preoperative optimization—encompassing weight management, stringent glycemic control, and smoking cessation—is imperative for mitigating the risk of postoperative infection.

### 3.2 Surgical‐Related Factors

Surgical technique, graft selection, and intraoperative protocols influence infection outcomes. Autografts remain the preferred choice due to their lower immunogenic potential [17, 18]. While autografts remain the preferred choice due to low immunogenicity, infection risks vary by type: retrospective evidence indicates higher infection rates in hamstring grafts compared with BPTB—potentially due to harvest‐related soft‐tissue trauma and smaller graft diameters [19, 20, 21]. Conversely, quadriceps tendon autografts demonstrate safety profiles comparable or superior to traditional BPTB [22]. Regarding allografts, while overall infection rates appear comparable under stringent sterilization, multicenter prospective data highlight elevated failure and infection risks specifically associated with irradiated grafts [23, 24, 25]. Additionally, synthetic LARS grafts offer biomechanical benefits, though their long‐term immunologic effects remain unclear [26].

Arthroscopic approaches yield lower infection rates than open procedures [6]. While double‐bundle techniques do not inherently increase risk [27, 28], prolonged operative time, multiple implants, and concomitant procedures (e.g., lateral extra‐articular tenodesis, LET) may elevate risk due to extended tissue exposure [29, 30, 31]. Nevertheless, LET appears to reduce revision risk in high‐demand populations [32]. Attention to sterile technique during graft preparation is also critical, as perioperative contamination remains a leading source of infection.

### 3.3 Medication‐Related Factors

The influence of corticosteroids on postoperative infection risk remains a subject of ongoing debate. Mechanistically, these agents suppress pro‐inflammatory cytokines such as IL‐1, TNF‐α, and prostaglandins to alleviate pain and swelling [33, 34]. However, this anti‐inflammatory benefit is coupled with broad immunosuppression—including impaired neutrophil migration and T‐cell dysfunction—which may compromise the host immune response. Clinical evidence regarding this risk remains heterogeneous. While some large‐scale retrospective cohorts and meta‐analyses report a significant increase in infections following perioperative or intra‐articular steroid exposure, particularly within 2 weeks of surgery [34, 35], other retrospective analyses have failed to establish a clear association [36]. This discrepancy may stem from differences in sample size, patient selection criteria, and the ability to control for confounding variables such as dosage and timing of administration. Beyond immunological concerns, corticosteroids may mask early clinical signs of infection, potentially delaying diagnosis. Furthermore, repeated intra‐articular injections may disrupt local tissue architecture and impair graft integration, theoretically increasing susceptibility to microbial colonization [37]. Given these concerns, the use of corticosteroids in ACLR must be carefully individualized, weighing short‐term symptomatic relief against the elevated risk in vulnerable subgroups, such as diabetic or revision patients.

Investigations into risk factors for infection following ACLR are constrained by heterogeneities in study design. While prospective cohorts are invaluable for tracking long‐term outcomes such as reinjury and functional recovery [38, 39], However, infection‐related outcomes are usually reported only as secondary findings, and event numbers are too low to allow robust statistical comparisons. Preventive measures such as graft presoaking with vancomycin have only recently begun to be tested prospectively, showing promising reductions in septic arthritis, though data remain limited and largely confined to single‐center studies. While prospective studies set a higher evidentiary standard, their scarcity underscores the need for complementary insights from retrospective research. A summary of representative clinical studies investigating infection management and treatment outcomes following ACL reconstruction over the past decade is presented in Table 1 [40, 41, 42, 43, 44, 45, 46, 47, 48].

**TABLE 1:** Summary of representative clinical and translational studies on infection management following ACLR in the past decade.

Study (year)	Design	N_total	N_infected	Follow‐up	Main outcomes	Source
Schuster P (2020)	Single‐centre cohort (retrospective control; prospective vancomycin group)	10,516 (control 8222; vancomycin 2294)	35 in control (0.4%); 0 in vancomycin group (0.0%)	Vancomycin presoaking reduced septic arthritis from 0.4% to 0.0%	Vancomycin presoaking reduced septic arthritis from 0.4% to 0.0%	[40]
Banios K et al. (2021)	Single‐centre before–after cohort	1835 (period1: 1242 no‐vancomycin; period2: 593 vancomycin)	7/1242 (0.56%) in period1; 0/593 (0.0%) in period2	Routine early postop follow‐up	Significant reduction to zero infections after adopting vancomycin presoaking (*p* = 0.018)	[41]
Carrozzo A et al.(2022)	Single‐centre cohort+ systematic review & meta‐analysis	Cohort 5300; Meta‐analysis 29,659 ACLR	Cohort: 11/3228 (0.34%) control vs. 1/2072 (0.05%) presoaked; Meta‐analysis: pooled OR 14.39 (95% CI 5.90–35.10) for no vancomycin	Study periods variable across included studies; cohort contemporaneous institutional series	Vancomycin presoaking associated with large reduction in septic arthritis; strong pooled effect	[42]
Lamplot JD (2021)	Laboratory biomechanical study + review	In vitro specimens (lab tests)	NA (biomechanical focus)	Time‐zero biomechanical testing	Vancomycin soaking does not adversely affect time‐zero graft material properties	[43]
Themessl A et al. (2022)	Single‐centre retrospective cohort of infected patients	38 infected patients included (44 eligible)	38 (study cohort)	Mean 60.3 ± 39.9 months	Lysholm 80.0; IKDC 78.2; median RTS 8 months; graft presence at FU improved IKDC; 62.2% returned to at least one pivoting sport	[44]
Tong K et al.(2022)	Animal model + small clinical retrospective series	Clinical *n* = 12 patients with DKI after ACLR	Clinical cohort: 12 patients (all treated)	6 months (clinical outcomes reported)	Surgical debridement + systemic vancomycin ± rifampin eradicated *S. aureus* in animal model; clinical series showed infection control in most patients at 6 month	[45]
Costa GG et al. (2021)	Retrospective diagnostic study	3408 ACLR screened; 24 infected +14 uninfected included (total 38)	24 confirmed infection (analyzed alongside 14 controls)	Diagnostic metrics at time of readmission; not a long‐term outcome study	Synovial WBC count most reliable marker; optimal cutoff ~28,100 cells/mL for highest accuracy	[46]
Eisenberg MT et al. (2022)	Large database retrospective	44,501 patients (< 18 years cohort)	Infection rate 0.52% (< 18 years)	Database perioperative window; outcomes focused on infection incidence	Low overall infection rate (~0.5%); slightly lower rates in < 15 years. (0.37%) compared with adolescents	[47]
Ma Y et al. (2024)	Single‐centre retrospective cohort	1500 ACLR between Jan 2011 and Jan 2022	20 patients meeting infection criteria summarized	Reported clinical course and treatment outcomes (per article)	Standardized treatment algorithm described; case series of 20 infections used to illustrate outcomes	[48]

*Note:* This table summarizes key studies investigating infection prevention and treatment strategies following ACL reconstruction (ACLR), including both prospective and retrospective cohorts. Quantitative outcomes (infection rates, follow‐up duration, and functional recovery scores) are presented when available.

Abbreviations: ACLR, anterior cruciate ligament reconstruction; DKI, deep knee infection; FU, follow‐up; IKDC, International Knee Documentation Committee score; NA, not applicable; RTS, return to sport; WBC, white blood cell.

## 4 Treatment Strategies

Management of infection after ACLR presents a multifaceted clinical challenge, requiring careful integration of surgical strategy, antimicrobial therapy, and patient‐specific considerations. The overall goal of treatment is to eradicate infection while preserving or restoring knee function and minimizing long‐term morbidity. Infection not only threatens graft integrity but can also result in significant complications such as arthrofibrosis, osteochondral damage, and eventual graft failure if not addressed promptly (Table 2).

**TABLE 2:** Summary of treatment strategies for infection following anterior cruciate ligament reconstruction.

Subsection	Key points	Clinical considerations	Common indications	Recommended timing	References
Treatment Principles and Decision Framework	Early diagnosis and immediate surgery + antibiotics are key. Graft removal depends on infection severity and patient factors	Assess patient comorbidities, signs of systemic infection, and MRI/arthroscopic findings to guide early intervention.	Indications: early infection, intact graft, stable fixation; Contraindications: severe contamination, necrotic graft.	Immediately after clinical diagnosis and positive synovial/blood culture.	[49, 50]
Surgical Management Pathways	Three main options: graft retention with debridement, delayed removal, or immediate removal. Early retention has high success	Evaluate graft appearance (intact vs. purulent), time since surgery, and response to initial debridement.	Indications: partial graft integrity, failed 1st debridement; Contraindications: rapid deterioration, osteomyelitis.	First 7 days for graft retention; beyond 2 failed debridements consider removal.	[51, 52]
Graft‐Related Complications and Subclinical Infections	Mild or hidden infections can damage grafts and cause bone issues. Long‐term effects still unclear	Monitor for persistent joint symptoms (pain/swelling), consider PCR or histology for low‐grade infection diagnosis.	Indications: persistent symptoms post‐ACLR, culture‐negative infection; Contraindications: unclear microbiological evidence.	Monitor post‐op for ≥ 6 weeks; consider diagnosis if delayed healing or pain.	[53, 54]
Timing of Revision ACLR	Delay revision for 3–6 months if bone infection exists. Without bone infection, may consider at 6 weeks if inflammation resolves	Use inflammatory markers (CRP, ESR), MRI signs of bone involvement, and prior surgical timeline to guide timing.	Indications: clinical resolution, normalized labs; Contraindications: unresolved infection, osteolysis, sinus tract.	6 weeks (no bone infection); 3–6 months (with osteomyelitis).	[55]
Antimicrobial Therapy	Start with broad‐spectrum antibiotics, then adjust based on cultures. Use drugs that target biofilm. Switch to oral if stable	Start empirical antibiotics immediately post‐debridement; modify based on antibiogram and patient renal/hepatic status.	Indications: confirmed bacterial growth, graft retention; Contraindications: allergy to key drugs, hepatic insufficiency.	IV for 1–2 weeks then oral for 4–6 weeks; transition based on clinical stability.	[56, 57, 58]

### 4.1 Treatment Principles and Decision Framework

Timely diagnosis followed by prompt initiation of combined surgical and antibiotic therapy remains the cornerstone of management [7, 49]. Once infection is confirmed, surgical irrigation and debridement combined with culture‐specific antibiotics constitute the standard therapeutic pathway. However, a key clinical dilemma often lies in whether to retain or remove the ACL graft [50]. While many surgeons favor graft preservation—especially in early‐detected or less severe infections—no universally accepted guidelines exist. Decisions are therefore individualized based on intraoperative findings, graft integrity, extent of contamination, and patient comorbidities.

### 4.2 Surgical Management Pathways

Three principal surgical strategies are described in the literature: [1] graft retention with one or multiple rounds of arthroscopic debridement, [2] delayed graft removal after failed debridement attempts, and [3] immediate graft removal [59, 60].

#### 4.2.1 Graft Retention and Arthroscopic Debridement

Most studies support initial graft retention when the graft appears structurally sound and contamination is limited. Konstantinou E et al. reported that, in such cases, surgical debridement combined with systemic antibiotic therapy yields graft preservation rates exceeding 80% and are associated with better postoperative function, reduced hospital stay, and lower complication rates [51].

The principle behind graft retention is that an intact and well‐fixed graft, if adequately irrigated and debrided, can serve its biomechanical function once the infection is eradicated. Several prospective and retrospective cohorts have demonstrated that early debridement, combined with biofilm‐active antibiotic regimens, can achieve infection resolution without the morbidity of staged reconstruction [61, 62]. In these cases, repeated arthroscopic washouts (often one to three procedures) are acceptable and may be necessary to achieve clearance. Functional outcomes after successful graft retention are generally superior to those after graft removal, particularly in young athletic populations.

#### 4.2.2 Indications for Delayed Graft Removal

Nevertheless, graft removal may become necessary in the setting of recurrent infection, fixation failure, purulent graft coating, or visible degeneration during arthroscopic assessment. Notably, GD Cassano et al. showed that failure to achieve infection resolution after two or more debridements often shifts the balance in favor of graft explantation [8].

The threshold for switching from retention to removal is not universally standardized, but most series suggest that two unsuccessful debridements should prompt consideration of removal to avoid prolonged morbidity. Osteomyelitis involving the tibial or femoral tunnels and atypical infections such as fungal or tubercular arthritis are other clear indications for removal. These complex cases may require open arthrotomy and extended antimicrobial therapy, underscoring the importance of individualized surgical planning.

#### 4.2.3 Immediate Graft Removal and Decision Determinants

Although immediate graft removal is less commonly performed, it may be warranted when infections are diagnosed late (> 7 days post‐ACLR) or when the graft is deemed irreparably compromised. Runer A et al. reported higher removal rates in delayed diagnosis scenarios or when prolonged antibiotic therapy has failed to yield improvement [52]. Other studies have emphasized that late‐presenting infections often involve biofilm maturation and deeper bone involvement, both of which significantly reduce the likelihood of successful graft retention [61]. In such situations, immediate graft removal with thorough tunnel debridement is advocated to minimize the risk of chronic osteomyelitis.

It is important to note, however, that immediate graft removal carries disadvantages, including the guaranteed need for a secondary reconstruction, potential for joint instability, and prolonged rehabilitation timelines. Some decision‐analytic models suggest that while removal reduces the risk of persistent infection, it increases the cumulative burden of future surgeries [63]. Therefore, treatment choice must balance infection eradication with patient function and quality of life. Shared decision‐making with the patient, incorporating clinical urgency, organism virulence, graft condition, and patient expectations, is essential to achieve optimal outcomes.

In summary, surgical pathways for infection after ACLR can be viewed as a continuum: initial graft preservation is justified in early, limited infections; delayed graft removal is indicated after failed attempts or in more aggressive presentations; and immediate graft removal is reserved for severe, late, or atypical infections. This tiered approach highlights the need for flexible, evidence‐informed decision‐making tailored to the individual patient.

### 4.3 Graft‐Related Complications and Subclinical Infections

Emerging research highlights the detrimental impact of subclinical, low‐grade infections—often associated with bacterial biofilm formation—on graft durability. Studies by Chittajallu demonstrated that these infections can weaken the collagen structure within the graft, thereby reducing its tensile strength and contributing to mechanical failure [53]. Moreover, David C. Flanigan found that increased detection of bacterial DNA in widened tibial tunnels suggests a possible correlation between persistent bacterial burden and tunnel osteolysis [54].

The role of biofilms is particularly concerning, as they allow pathogens such as coagulase‐negative staphylococci and Cutibacterium acnes to persist at low metabolic activity, evading host immune surveillance and standard culture techniques. Such organisms may colonize graft fibers, fixation devices, or bone tunnels without causing overt clinical symptoms, yet progressively compromise graft integrity. Animal models and histological analyses have demonstrated matrix metalloproteinase activation and chronic inflammatory cell infiltration around colonized grafts, leading to gradual weakening of tendon‐bone integration [64]. Clinically, patients with subclinical infections may present with nonspecific symptoms such as persistent effusion, delayed graft incorporation, or unexplained tunnel widening rather than overt septic arthritis. These findings are often mistaken for mechanical graft failure or “biological non‐integration.” Advanced molecular techniques, including PCR and next‐generation sequencing (NGS), have significantly improved the detection of bacterial DNA in cases previously deemed culture‐negative [54]. Such techniques suggest that subclinical bacterial persistence may be more common than previously recognized. The long‐term implications of these subclinical infections include compromised graft strength, increased risk of tunnel osteolysis, and higher likelihood of revision surgery. A number of registry‐based studies report that patients with unexplained tunnel widening or delayed graft failure often show molecular evidence of bacterial colonization [65]. This raises the possibility that a subset of so‐called “aseptic failures” is in fact infection‐related. From a therapeutic perspective, early recognition of subclinical infections is challenging but clinically important. Strategies under investigation include prophylactic graft presoaking with vancomycin or gentamicin, local antimicrobial coatings on fixation devices, and adjunctive therapies targeting biofilm disruption [66]. Further prospective studies are needed to clarify diagnostic criteria, identify reliable biomarkers, and determine whether targeted antimicrobial therapy can improve graft survival in these cases.

In summary, subclinical infections represent a “hidden threat” to graft longevity after ACL reconstruction. Although their diagnosis remains difficult, accumulating evidence suggests they may contribute significantly to graft weakening, tunnel osteolysis, and eventual failure. Recognizing their potential role underscores the importance of continued innovation in both preventive strategies and advanced diagnostic modalities.

### 4.4 Timing of Revision ACLR

For patients requiring graft removal, the timing of revision ACLR must be carefully determined. Most guidelines, including those from ESSKA and EBJIS, recommend a waiting period of 3 to 6 months when osteomyelitis is present [55]. In cases without bone involvement, revision reconstruction may be considered as early as 6 weeks post‐treatment, provided that the infection has resolved clinically, CRP has normalized, and inflammatory signs have abated. Graft selection during revision should be approached cautiously, with consideration of prior infection risk factors and surgical history. Allografts may carry a slightly higher theoretical risk in previously infected joints, and this should be factored into graft choice.

### 4.5 Antimicrobial Therapy

Antibiotic management remains a vital adjunct to surgical treatment [51]. In the empirical phase, broad‐spectrum coverage is appropriate until culture results are available; typical choices include a beta‐lactam/beta‐lactamase inhibitor backbone plus vancomycin or daptomycin to cover Gram‐positive cocci including methicillin‐resistant *Staphylococcus aureus* and coagulase‐negative staphylococci [56] Once culture results become available, targeted therapy should be adopted. If hardware or graft material is retained, incorporation of biofilm‐active agents such as rifampin is strongly recommended especially for staphylococcal infections paired with fluoroquinolones for optimal synergy [57]. Rifampin should only be started after drains have been removed and the wound is dry, to avoid resistance development. The typical duration of therapy ranges from 4 to 6 weeks, with early transition to oral therapy feasible in stable patients. Oral agents should be bactericidal, possess high bioavailability, and demonstrate good penetration into joint and bone tissues. Scarborough M et al. recently reported that the growing trend toward shorter intravenous therapy durations is supported by recent studies in bone and joint infections, suggesting that carefully monitored oral step‐down protocols are both effective and safe [58]. Supporting this, a more recent consensus article on antimicrobial therapy in implant‐associated infections underscores that antibiotics should be tailored based on pharmacokinetics/pharmacodynamics, bone penetration, and biofilm considerations (e.g., rifampin combinations) [67].

### 4.6 Antibiotic Management

Antibiotic management is central to both the prevention and treatment of infections following ACLR. In recent years, intraoperative graft presoaking with antibiotics, particularly vancomycin, has emerged as a widely supported strategy to reduce postoperative infection risk [68]. A meta‐analysis conducted by Figueroa F et al. involving thousands of patients demonstrated a dramatic reduction in septic arthritis rates with vancomycin presoaking, typically using a 5 mg/mL solution for 10–15 min prior to graft implantation. Concerns about cytotoxicity have largely been alleviated by pharmacological studies showing intra‐articular concentrations remain far below toxic thresholds and biomechanical assessments confirming no negative impact on graft strength. Gentamicin has also been explored as an alternative, especially for broader Gram‐negative coverage, although high‐quality comparative studies are currently lacking [69].

Standard perioperative protocols continue to emphasize timely systemic antibiotic administration [70]. Prophylaxis usually consists of a single intravenous dose of cefazolin or cefuroxime given within 1 h prior to incision. In patients with documented beta‐lactam allergies or MRSA colonization, vancomycin serves as a recommended substitute. Notably, prolonged antibiotic use beyond 24 h has not been shown to provide additional benefit and may increase the risk of resistance or adverse events. When postoperative infection is suspected, antibiotics should be initiated promptly after diagnostic aspiration of joint fluid [71]. Empirical therapy typically combines coverage against both Gram‐positive and Gram‐negative organisms using agents such as a beta‐lactam/beta‐lactamase inhibitor plus vancomycin or daptomycin. Once microbiological data are available, therapy should be adjusted to target the identified pathogen. In cases involving retained grafts or implants, the addition of biofilm‐active agents like rifampin, often paired with fluoroquinolones, has been shown to improve outcomes, especially in infections caused by *Staphylococcus aureus* or Cutibacterium acnes.

The duration of antibiotic treatment varies based on the clinical scenario, but current practice generally involves an initial intravenous phase lasting one to 2 weeks, followed by oral therapy for an additional 4 to 5 weeks. In complex cases—particularly those involving biofilms or retained foreign material—longer courses may be necessary [69]. Clinical improvement and normalization of inflammatory markers, especially C‐reactive protein, are key indicators guiding the transition from intravenous to oral therapy. Multidisciplinary input, including consultation with infectious disease specialists, is often essential for optimizing individualized antibiotic regimens and ensuring effective eradication of infection while preserving joint function.

## 5 Stratified Management Strategies in Different Age Groups

Postoperative infections after anterior cruciate ligament reconstruction require individualized management strategies, especially when accounting for age‐related differences in physiological responses and recovery capabilities. Although both adolescents and older adults are at risk for infections, their distinct stages of development and underlying health conditions demand different treatment approaches.

### 5.1 Management Strategies in the Adolescent Population

The incidence of post‐operative infection following anterior cruciate ligament (ACL) reconstruction in the pediatric and adolescent population is relatively low, at ~0.52%, which is comparable to that observed in young adults [47]. Despite this low prevalence, the impact of infection on skeletal growth and long‐term functional outcomes in adolescents remains a critical concern. Given that adolescents are in a pivotal stage of skeletal and soft tissue maturation, potential physeal injury may lead to severe complications, including angular deformities or limb‐length discrepancies, thereby interfering with normal bone development and joint functional recovery. Evidence suggests that post‐infectious arthrofibrosis and functional impairment can significantly delay the return‐to‐sport timeline and compromise long‐term athletic performance [72]. Furthermore, infection may impede graft healing or lead to graft failure, increasing the risk of subsequent knee reinjury. Consequently, therapeutic strategies for postoperative infection in adolescent patients must simultaneously prioritize both infection eradication and the preservation of growth potential.

The cornerstone of management lies in early recognition and emergent intervention. Upon diagnosis, arthroscopic debridement combined with continuous joint irrigation should be performed within 24 to 48 h. During the procedure, a posteromedial approach is recommended to safeguard the blood supply to the physis and minimize the risk of iatrogenic growth plate injury [73]. Whenever feasible, every effort should be made to retain the graft to maintain joint stability; however, graft excision must be considered if the graft itself is grossly involved in the infectious process. For infectious foci within the bone tunnels, the localized implantation of antibiotic‐impregnated bone cement beads may be utilized to achieve high regional concentrations of antimicrobial agents.

Beyond clinical intervention, the adolescent population requires comprehensive psychological and social support to facilitate a successful return to sports and the resumption of normal daily activities.

### 5.2 Management Strategies in the Elderly Population

With the aging population and the increasing popularity of physical exercise, the number of individuals over the age of 50 undergoing anterior cruciate ligament reconstruction has risen annually. Although clinical score improvements in this cohort are comparable to those in younger patients, the graft failure rate is significantly higher (17.6% vs. 6.5%) [74, 75]. While the incidence of postoperative infection (POI) is slightly lower in the elderly, age‐related immunosenescence renders infections more prone to systemic dissemination, potentially leading to sepsis, prolonged recovery, and an elevated risk of revision surgery [76, 77]. POI not only delays rehabilitation but also precipitates long‐term functional impairment. Compromised bone and soft tissue healing in elderly patients, exacerbated by infection, can trigger arthrofibrosis, muscle atrophy, and permanent loss of motor function, markedly diminishing quality of life. Due to the atypical clinical presentation of infection in this population, therapeutic strategies should prioritize the prevention of systemic complications and the early identification of latent infections. Furthermore, given the age‐related decline in metabolic capacity and the associated risk of drug accumulation, the selection and dosage of antibiotics must be meticulously calibrated to minimize adverse effects. Finally, as elderly patients frequently present with multimorbidity (e.g., diabetes, hypertension), the management of POI must be integrated with the comprehensive control of underlying diseases to optimize overall clinical outcomes.

## 6 Prevention and Rehabilitation Strategies

Following the successful eradication of infection after ACL‐R, restoring functional stability and enabling patients to return to daily life or athletic activity becomes a central objective of long‐term care [44]. However, postoperative infections, particularly septic arthritis, are associated with increased risk of joint stiffness, cartilage damage, graft dysfunction, and ultimately impaired clinical outcomes. Therefore, a well‐structured rehabilitation and prevention strategy is crucial to optimize recovery. Long‐term follow‐up studies have revealed that functional outcomes after infection are generally inferior to those observed in standard ACLR cohorts. In a prospective study with a mean follow‐up of 6 years, Gille et al. reported a significant decrease in activity levels among 31 patients who had previously experienced infection [78]. The mean Tegner score dropped from 6.5 preoperatively to 4.5 post‐treatment, with an average Lysholm score of 63.9 and a subjective IKDC score of 63. These results underscore the lingering impact of infection on knee function. Similarly, in a retrospective case series with a minimum follow‐up of 2 years, patients achieved mean Lysholm and IKDC subjective scores of 80.0 and 78.2, respectively, but more than half of the patients (55.3%) had ceased sporting activity, and over 50% reported reduced frequency of participation compared with their pre‐injury status [44]. Only 26.3% successfully returned to sport at their pre‐injury frequency, highlighting the challenges associated with full recovery.

Interestingly, Themessl A et al. found that return to sports (RTS) rates did not significantly differ depending on the initial graft management approach, whether the graft was retained during infection management or explanted and subsequently revised [44]. This suggests that functional outcomes may be more closely linked to other factors, such as timing of intervention, rehabilitation quality, and individual patient response. Indeed, persistent ACLR deficiency at follow‐up was the only patient‐dependent factor consistently associated with lower RTS rates. Two additional case–control studies—conducted by Abdel‐Azis et al. [79] and Bostrom Windhamre et al. [80] reported RTS rates of 56% and 62.5%, respectively, primarily for recreational activities, without specifying rates for return to competitive sports. Given these findings, prevention of infection and optimization of rehabilitation are critical. Preoperative measures include patient counseling on modifiable risk factors. Obesity and smoking are known to impair wound healing and immune response; therefore, weight reduction and smoking cessation are strongly recommended prior to surgery. Surgical site preparation with proper antiseptic techniques, including the use of chlorhexidine‐based skin cleaning and intraoperative antisepsis, is essential. Intraoperatively, unnecessary complexity should be avoided to reduce exposure time and tissue damage. Carrozzo A et al. reported that the use of autografts, particularly bone‐patellar tendon‐bone (BPTB) grafts, is associated with a lower infection risk [42]. Soaking grafts in vancomycin prior to implantation has demonstrated a significant reduction in infection rates and is increasingly adopted as a preventive standard.

Postoperatively, early and individualized rehabilitation plays a pivotal role in preventing complications such as arthrofibrosis and joint stiffness, which are major contributors to long‐term functional limitation [81]. Rehabilitation should be initiated once infection is controlled, based on the patient's tolerance and joint status. A gradual protocol that emphasizes restoration of range of motion, quadriceps and hamstring strengthening, and neuromuscular control is recommended. Careful progression of activity with attention to signs of joint inflammation or effusion is crucial. The use of cryotherapy, manual therapy, and structured physiotherapy may aid in symptom control and function restoration. Additionally, patients must be advised to avoid contact with high‐risk environments or contaminated surfaces during the recovery phase. Regular outpatient follow‐up is critical to detect signs of reinfection early and to reassess the rehabilitation trajectory. For immunocompromised individuals or those with recurrent infections, closer monitoring and extended prophylactic strategies may be necessary.

## 7 Conclusion and Future Directions

Postoperative infection after ACL reconstruction, though rare, remains a serious complication that threatens graft integrity and long‐term knee function. Current evidence emphasizes that timely surgical debridement combined with targeted antimicrobial therapy is central to successful management, while preventive strategies such as vancomycin presoaking and strict aseptic technique further reduce risk. Recent clinical data—both prospective and retrospective—support graft preservation in early, well‐controlled infections, whereas revision or staged reconstruction is reserved for refractory cases. Remaining challenges include the recognition of low‐grade and biofilm‐associated infections and the lack of standardized treatment protocols. Future research should prioritize multicenter prospective studies and meta‐analyses to refine therapeutic algorithms, optimize antibiotic duration, and improve long‐term functional outcomes through individualized rehabilitation.

## Author Contributions

S.Z. conceived the study, defined its scope, conducted the literature search, and drafted the manuscript. J.G. organized the thematic structure and revised the content. All authors participated in manuscript preparation and approved the final submitted version.

## Funding

This work was supported by the Dalian Medical Science Research Program (Grant No. 2311038) and Basic Research Projects of Colleges and Universities in Liaoning Province: LJ222511258004.

## Disclosure

All authors listed meet the authorship criteria according to the latest guidelines of the International Committee of Medical Journal Editors (ICMJE). All authors have reviewed and approved the final version of the manuscript and agree to be accountable for all aspects of the work.

## Ethics Statement

The authors have nothing to report.

## Consent

The authors have nothing to report.

## Conflicts of Interest

The authors declare no conflicts of interest.

## Data Availability

Data sharing not applicable to this article as no datasets were generated or analysed during the current study.
